# Use of Laser Speckle Contrast Imaging for Distribution of Animals by Severity of Brain Tissue Damage in a Neonatal Hypoxia-Ischemia Model in Mice

**DOI:** 10.3390/brainsci16010102

**Published:** 2026-01-17

**Authors:** Vladimir Pokrovskii, Konstantin Lapin, Viktoria Antonova, Mikhail Korokin, Oleg Gudyrev, Vladimir Gureev, Liliya Korokina, Olesya Scheblykina, Arkadii Nesterov, Maria Maslinikova, Ivan Chatsky, Denis Mukhamedov, Mikhail Pokrovskii

**Affiliations:** 1 Federal State Autonomous Educational Institution of Higher Education, Belgorod National Research University, Pobedy St., 85 1, 308015 Belgorod, Russia; mkorokin@mail.ru (M.K.); gudyrev@mail.ru (O.G.); produmen@yamdex.ru (V.G.); korokina@bsuedu.ru (L.K.); shcheblykina@bsuedu.ru (O.S.); nesterov_a@bsuedu.ru (A.N.); maria.maslinikova@gmail.com (M.M.); 1212327@bsuedu.ru (I.C.); nevrologia31@inbox.ru (D.M.); pokrovskii@bsuedu.ru (M.P.); 2 Research Institute of General Reanimatology Named After V. A. Negovsky Federal Scientific and Clinical Center of Reanimatology and Rehabilitation, Petrovka Street, 25, Building 2, 107031 Moscow, Russia; k.n.lapin@gmail.com (K.L.); victoryant.sci@gmail.com (V.A.); 3 Federal State Budgetary Institution National Medical Research Center of Cardiology Named After Academician E.I. Chazov, Akademika Chazova St., 15A, 121552 Moscow, Russia

**Keywords:** neonatal hypoxia–ischemia, laser speckle contrast imaging, cerebral perfusion, injury severity stratification, preclinical mouse model, experimental variability

## Abstract

**Background/Objectives**: Inter-individual variability in injury severity represents a major barrier to reproducibility in neonatal hypoxia–ischemia (HI) models. Objective early postoperative stratification of animals is therefore essential for standardized group allocation and reliable assessment of experimental outcomes. This study aimed to evaluate whether laser speckle contrast imaging (LSCI) can be used as a rapid, noninvasive tool for early post hoc stratification of ischemic brain damage severity in neonatal mice following HI. **Methods**: Neonatal CD-1 mice (postnatal day 9; *n* = 60) underwent hypoxia–ischemia using a modified Rice–Vannucci protocol. Cerebral perfusion was assessed by laser speckle contrast imaging at baseline, 3 h, and 7 days after HI. The difference in mean perfusion between ipsilateral and contralateral hemispheres at 3 h (Δ perfusion) was used to stratify animals into severity groups. Brain injury was quantified by 2,3,5-triphenyltetrazolium chloride (TTC) staining at 24 h and 7 days. Survival was monitored for 7 days and analyzed using Kaplan–Meier curves and the log-rank (Mantel–Cox) test. **Results**: LSCI-derived Δ perfusion at 3 h enabled the formation of distinct injury-severity groups (no visible damage, mild, moderate, and severe) with significant between-group differences (*p* < 0.0001). TTC-based lesion area increased stepwise across severity groups, and Δ perfusion correlated with lesion size when all animals were analyzed together (r = 0.688, *p* = 0.0011). No significant correlations were observed within individual severity groups, indicating that the overall association was driven primarily by between-group differences. Survival analysis revealed 75% mortality in the severe injury group (*p* < 0.0001). **Conclusions**: LSCI represents a robust and practical approach for early, objective, group-level stratification of neonatal mice by HI injury severity, thereby improving reproducibility and statistical validity in preclinical studies. However, its ability to predict outcomes within individual severity categories is limited, and repeated long-term measurements may pose technical challenges.

## 1. Introduction

Neonatal hypoxia–ischemia (HI) remains one of the leading causes of neonatal mortality and long-term neurological disability, including cerebral palsy, epilepsy, and cognitive impairment [1]. Despite advances in perinatal care, effective neuroprotective strategies remain limited, and translational success from preclinical studies has been inconsistent [2,3]. Experimental rodent models of neonatal HI, particularly the Rice–Vannucci model, therefore remain indispensable for elucidating injury mechanisms and for preclinical evaluation of candidate therapeutic interventions [4,5].

A major challenge in neonatal HI research is the pronounced inter-individual variability in the extent and severity of brain injury observed following an identical ischemic insult. This variability arises from multiple factors, including strain-dependent differences in cerebrovascular anatomy, collateral circulation, metabolic maturity, and the heterogeneity of inflammatory and secondary injury responses [6,7]. As a consequence, animals with markedly different injury severities are often inadvertently assigned to the same experimental group, reducing statistical power, increasing variability of outcome measures, and contributing to poor reproducibility across studies.

To address this limitation, there is a critical need for objective, rapid, and noninvasive methods that allow early postoperative stratification of animals according to injury severity prior to therapeutic intervention. Conventional approaches for injury assessment, such as histological analysis or 2,3,5-triphenyltetrazolium chloride (TTC) staining, require animal sacrifice and therefore cannot be used for longitudinal evaluation or for early group allocation. Magnetic resonance imaging (MRI) provides high-resolution volumetric assessment of ischemic lesions but is expensive, low-throughput, and often inaccessible for routine use in preclinical laboratories [8,9,10,11].

Laser speckle contrast imaging (LSCI) is an optical imaging technique that enables real-time, wide-field assessment of relative tissue perfusion based on the analysis of speckle pattern fluctuations generated by coherent laser illumination of moving red blood cells [12]. LSCI offers several practical advantages for preclinical research, including high temporal resolution, minimal invasiveness, and the ability to capture spatially resolved perfusion maps over large cortical areas. The method has been widely validated for monitoring cerebral blood flow in experimental stroke models and for intraoperative and perioperative perfusion assessment [13,14,15,16,17].

Although LSCI has been extensively applied to characterize cerebral hemodynamics, its potential utility as a quantitative tool for early severity stratification in neonatal HI models has not been systematically investigated. In particular, whether early postoperative perfusion deficits measured by LSCI can be used to define reproducible, threshold-based injury severity categories—and thereby improve experimental group allocation and outcome interpretation—remains unclear.

In the present study, we investigated the feasibility of using LSCI-derived perfusion differences between the ipsilateral and contralateral hemispheres as an early, objective metric for stratifying neonatal mice by the severity of hypoxic–ischemic brain injury. Using a modified Rice–Vannucci model in postnatal day 9 mice, we established perfusion-based severity categories shortly after HI induction and validated this stratification using macroscopic TTC-based lesion assessment and survival outcomes. By providing a practical framework for early severity classification, this approach aims to reduce experimental variability and enhance the reproducibility and statistical validity of preclinical studies in neonatal hypoxic–ischemic brain injury.

Objective: To evaluate the feasibility of using early postoperative laser speckle contrast imaging as a noninvasive tool for post hoc stratification of neonatal mice according to hypoxic–ischemic brain injury severity and to validate this stratification using macroscopic tissue damage and survival outcomes.

## 2. Materials and Methods

### 2.1. Animals

All procedures involving animals were approved by the Institutional Animal Care and Use Committee of Belgorod National Research University (approval no. 01-08i/25, 18 August 2025). The ethical approval covered the experimental procedures and surgical interventions described in this study and did not include colony establishment or breeding, which were conducted independently of the present work. All efforts were made to minimize animal suffering and to reduce the number of animals used.

Neonatal outbred CD-1 mice (*n* = 60; postnatal day 9, P9) of both sexes were used in this study. All animals underwent hypoxia–ischemia (HI) surgery. Following HI induction, animals were subjected to early postoperative assessment using laser speckle contrast imaging and were subsequently stratified post hoc into injury severity groups based on perfusion differences between hemispheres.

Animals were included in downstream analyses according to data availability for each outcome measure (LSCI, TTC-based macroscopic assessment, and survival). The exact number of animals contributing to each analysis is indicated in the corresponding figure legends.

### 2.2. Experimental Design and Group Allocation

Animals were subjected to hypoxia–ischemia according to the same experimental protocol and were not assigned to predefined injury severity groups prior to the procedure. Injury severity groups were defined post hoc based on early postoperative laser speckle contrast imaging measurements obtained 3 h after hypoxia–ischemia.

Stratification was performed using the difference in mean perfusion between the ipsilateral and contralateral hemispheres, as described below. Animals exhibiting minimal perfusion asymmetry and no macroscopic evidence of tissue injury were classified as no-visible-damage (NVD) and were used as a physiological reference group.

Based on Δ perfusion, four stratification categories were formed: intact animals (non-operated animals, *n* = 6), no-visible-damage (NVD, *n* = 12), mild (*n* = 16), moderate (*n* = 15), and severe (*n* = 11). The NVD group was used exclusively as a physiological reference for validating the stratification method and was not included in subsequent analyses of outcomes (survival, tissue damage).

The number of animals included in each longitudinal assessment is specified below. These numbers reflect the post hoc stratification design, planned subsampling for terminal endpoints, and attrition due to mortality or technical constraints (e.g., wound healing preventing repeated LSCI):

- TTC staining at 24 h: mild group (*n* = 6), moderate group (*n* = 6), severe group (*n* = 4), intact controls (*n* = 3). NVD (*n* = 6)
- Survival monitoring over 7 days: mild (*n* = 10), moderate (*n* = 9), severe (*n* = 7), intact (*n* = 3).
- LSCI at 7 days: mild (*n* = 7), moderate (*n* = 7), severe (*n* = 2), intact (*n* = 3). Not all survivors from the mild and moderate groups could be re-imaged due to scalp wound healing.
- TTC staining at 7 days: mild (*n* = 10), moderate (*n* = 9), severe (*n* = 2), intact (*n* = 3). This analysis included all available brains at the endpoint.

### 2.3. Modeling of Neonatal Hypoxia–Ischemia

Neonatal hypoxia–ischemia (HI) was induced using a modified Rice–Vannucci method in 9-day-old CD-1 mouse pups [6,18]. Under isoflurane anesthesia (4% induction, 1% maintenance), the left common carotid artery was coagulated. After recovery, pups were returned to the dam for two hours and then exposed to hypoxic conditions (10% O_2_, 90% N_2_) for 50 min in a hypoxia chamber accommodating no more than 25 animals per session. Gas composition was continuously monitored using a PKG-4 V-K-P (Ecsis, Moscow, Russia) (62615-15 until 12 November 2025, Russia) gas analyzer.

### 2.4. Laser Speckle Contrast Imaging

Cerebral perfusion was assessed by LSCI using the RFLSI-ZW system (RWD Life Science, Shenzhen, China) 3 h after hypoxia–ischemia, prior to any outcome-based evaluation, under light isoflurane anesthesia, a midline scalp incision was made, and the skin was retracted to expose the skull surface for wide-field imaging.

For each LSCI image, data acquisition was performed using standardized imaging settings. Camera gain was set to 118; exposure time was 20 ms; and the raw sequence was recorded at a frame rate of 50 fps. Flux maps were generated using a spatial algorithm in sliding mode, with a filter constant of 15 s and a background threshold of 10. Both flux and color images were acquired at a resolution of 1032 × 772 pixels. The distance from the imaging head/camera to the imaging plane was kept constant at 146 mm. The field of view corresponded to an image size of 17.62 × 13.18 mm, ensuring comparable spatial sampling and subsequent quantitative processing across animals and time points. Mean perfusion was calculated within a predefined rectangular region of interest (ROI) measuring 6.1 mm (length) × 2.1 mm (width); the ROI was positioned consistently across images at 2 mm from the sagittal suture and 1 mm from the coronal suture.

Mean perfusion values were quantified separately for the ipsilateral (injured) and contralateral hemispheres, and the difference between hemispheres (Δ mean perfusion) was calculated for each animal. Regions of interest (ROIs) corresponding to the ipsilateral and contralateral hemispheres were manually defined, and mean perfusion values were extracted using dedicated analysis software. Perfusion asymmetry was calculated as the difference in mean perfusion units between hemispheres [16,17]:

$$\Delta MP=ROI^{1}-ROI^{2}$$

ΔMP: mean perfusion delta; ROI^1^: mean perfusion value within the selected region in the contralateral hemisphere; ROI^2^: mean perfusion value within the selected region in the ipsilateral (injured) hemisphere.

Animals were stratified post hoc into injury severity categories no-visible-damage, mild, moderate, severe based solely on Δ mean perfusion values obtained at this early time point. These categories were subsequently used for group-based analyses of macroscopic brain injury and survival outcomes.

Injury severity thresholds were defined empirically based on the distribution of perfusion asymmetry values obtained across the experimental cohort. Thresholds were selected to reflect increasing degrees of hemispheric perfusion deficit and were used exclusively for within-study stratification rather than as universal criteria.

### 2.5. Post-Traumatic Assessment

Mortality data were analyzed using Kaplan–Meier survival curves to evaluate survival dynamics within 7 days after HI induction. Differences among severity groups (no lesion, mild, moderate, severe) were assessed using the log-rank test (*p* < 0.05).

### 2.6. Euthanasia of Animals

Decapitation was used as a method of euthanasia for neonatal mice, in accordance with the AVMA Guidelines for the Euthanasia of animals and as approved by the Institutional Animal Care and Use Committee (IACUC). All procedures were performed by trained personnel using sharp instruments to ensure rapid loss of consciousness and minimize distress.

### 2.7. Macroscopic Evaluation of Brain Damage

To validate LSCI-based stratification, macroscopic brain injury was assessed 24 h after hypoxia–ischemia in a subset of animals. Coronal brain sections (1 mm thickness) were obtained using a mouse brain matrix (Brain Matrix-Mouse/40–75 g/Coronal/1 mm, RWD Life Science, Shenzhen, China), and sections from comparable rostro-caudal levels were selected for TTC staining (2% 2,3,5-triphenyltetrazolium chloride (TTC), Sigma-Aldrich, Saint Louis, MO, USA) to minimize anatomical variability.

The hemispheric area difference was quantified by pixel-based analysis using QuPath 0.4.3 software [19]. Animals classified as no-visible-damage (NVD) were included in this analysis as a physiological reference but were excluded from subsequent outcome-based analyses due to the absence of detectable macroscopic injury. To compare the extent of macroscopic injury between groups, the lesion metric was calculated as the difference between the summed tissue areas of the ipsilateral (injured) and contralateral hemispheres. The analysis was performed on serial 1 mm-thick coronal sections: six sections per animal collected 24 h after HIE induction and seven sections per animal collected 7 days after HIE induction. In the figures, one representative second section from each group is shown, starting at the level of the tentorium cerebelli.

### 2.8. Statistical Analysis

Statistical analyses were performed using GraphPad Prism Software 8.0 (GraphPad Software, San Diego, CA, USA). Normality was assessed using the Shapiro–Wilk test. Due to unequal variances and group sizes, Welch’s one-way ANOVA followed by Games–Howell post hoc testing was applied where appropriate. Nonparametric tests were used when assumptions for parametric analysis were not met. Data are presented as mean ± SD, and *p* < 0.05 was considered statistically significant.

## 3. Results

### 3.1. Randomization of Animals by Severity in the Acute Period

**Figure 1:**
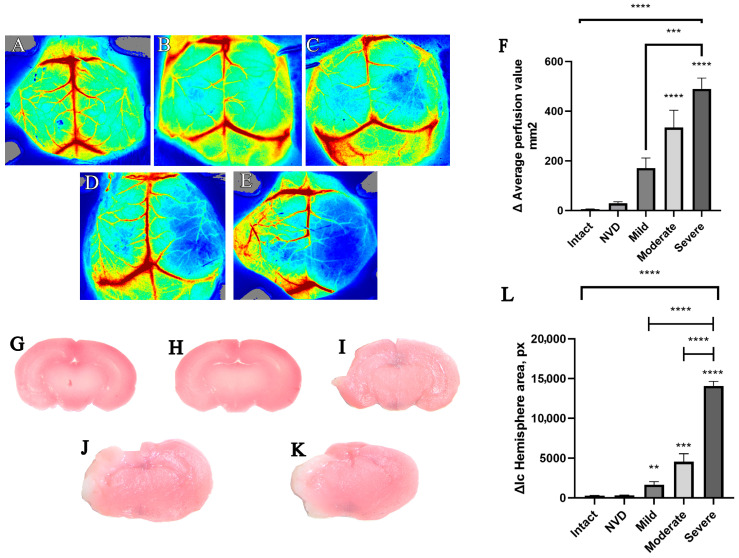
LSCI-based stratification of neonatal mice by severity of hypoxic–ischemic brain injury. Representative laser speckle contrast imaging maps obtained 3 h after hypoxia–ischemia are shown for (**A**) intact animals (*n* = 6), (**B**) no-visible-damage (NVD) group (*n* = 12), (**C**) mild (*n* = 16), (**D**) moderate (*n* = 15), and (**E**) severe injury (*n* = 11) groups. (**F**) Quantification of the difference in mean perfusion (Δ perfusion units) between ipsilateral and contralateral hemispheres across groups. Representative TTC-stained coronal brain sections obtained 24 h after hypoxia–ischemia are shown for (**G**) intact (n = 3), (**H**) NVD (*n* = 6), (**I**) mild (*n* = 6), (**J**) moderate (*n* = 6), and (**K**) severe injury (*n* = 4) groups. All sections were selected from comparable rostro-caudal levels. (**L**) Quantification of hemispheric area difference based on pixel analysis of TTC-stained sections. Data are presented as mean ± SD. Statistical significance was assessed using Kruskal–Wallis or Welch’s ANOVA with appropriate post hoc tests. ** *p* < 0.05, ***p < 0.001, **** *p* < 0.0001.

Based on laser speckle contrast imaging measurements obtained 3 h after hypoxia–ischemia (HI), neonatal mice were stratified according to the difference in mean perfusion between the ipsilateral and contralateral hemispheres. Representative LSCI perfusion maps for intact animals and for the no-visible-damage (NVD), mild, moderate, and severe injury groups are shown in Figure 1A–E.

Quantitative analysis revealed a stepwise increase in Δ mean perfusion across the severity spectrum (Figure 1F). Animals classified as NVD exhibited perfusion differences close to physiological baseline values (29 ± 6 perfusion units), comparable to intact controls. In contrast, progressively larger perfusion deficits were observed in the mild (171 ± 40), moderate (334 ± 69), and severe (490 ± 43) injury groups. Nonparametric analysis using the Kruskal–Wallis test demonstrated significant differences among groups (H(4) = 55.49, *p* < 0.0001). Post hoc Dunn’s multiple comparisons test confirmed significant differences between intact and moderate or severe groups (*p* < 0.0001), as well as between mild and severe groups (*p* = 0.0005), whereas no significant difference was detected between intact and NVD animals (*p* > 0.9999).

To validate LSCI-based stratification, macroscopic assessment of brain injury was performed 24 h after HI using TTC staining of coronal brain sections obtained at comparable rostro-caudal levels (Figure 1G–K). Representative sections demonstrated a clear increase in lesion extent with increasing injury severity, while intact and NVD brains showed no visible ischemic damage.

Quantification of hemispheric area differences confirmed these observations (Figure 1L). Mean lesion area increased in a stepwise manner from the mild (1756 ± 370 px) to moderate (4560 ± 992 px) and severe (14,096 ± 559 px) groups, whereas intact (290 ± 36 px) and NVD (319 ± 49 px) animals did not differ significantly. Due to heterogeneity of variances across groups (Brown–Forsythe test, *p* < 0.05), differences were analyzed using Welch’s one-way ANOVA, which revealed a significant overall group effect (W(4, 8.763) = 534, *p* < 0.0001). Post hoc Games–Howell tests indicated significant differences between all injury severity groups (*p* < 0.01), except for the comparison between intact and NVD animals (*p* = 0.8424).

Based on the absence of detectable macroscopic injury and the minimal perfusion differences observed, animals classified as NVD were excluded from subsequent outcome analyses and used only as a physiological reference group.

### 3.2. Survival Analysis Following Severity-Based Stratification

**Figure 2:**
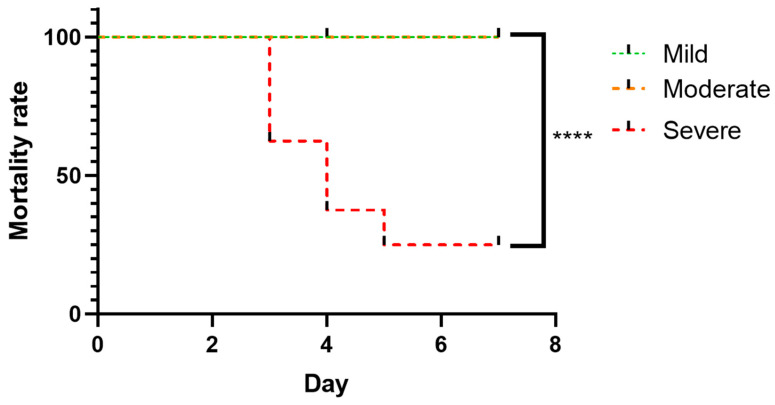
Survival analysis following early postoperative severity-based stratification. Kaplan-Meier curve format; **** *p* < 0.0001.

Survival was assessed as an additional outcome measure in animals stratified by early postoperative LSCI-based injury severity. Kaplan–Meier survival analysis revealed marked differences among injury severity groups (Figure 2).

Animals classified as having severe injury exhibited a high mortality rate (75%), with the first lethal event occurring on day 3 after hypoxia–ischemia. In contrast, no mortality was observed in the mild and moderate injury groups during the observation period. Log-rank (Mantel–Cox) analysis demonstrated a significant difference in survival among groups (χ^2^ = 21.41, df = 2, *p* < 0.0001).

Animals classified as no-visible-damage were not included in survival analysis due to the absence of detectable injury and lack of mortality events.

### 3.3. Delayed Assessment at Day 7 of Cerebral Perfusion and Brain Damage

**Figure 3:**
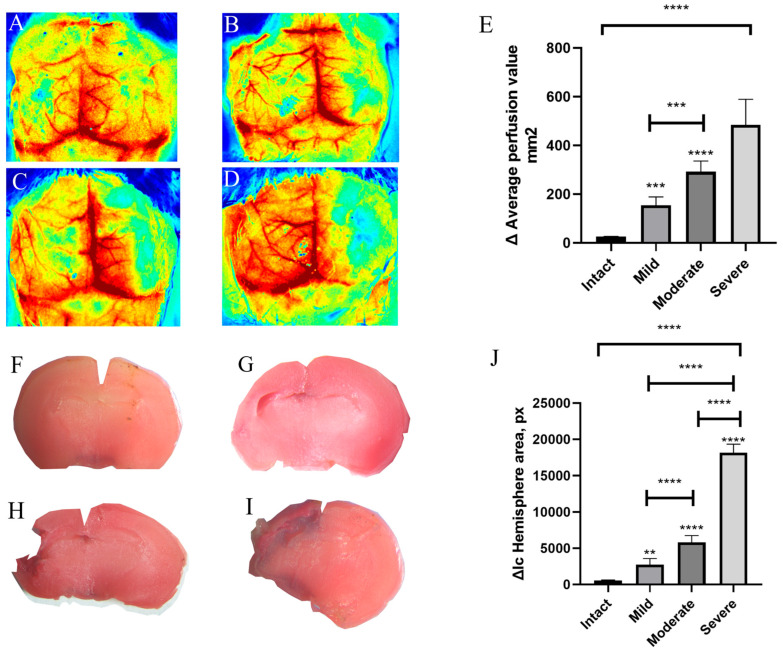
Assessment of cerebral perfusion and macroscopic brain injury 7 days after hypoxia–ischemia in severity-stratified groups. (**A**–**D**) Representative laser speckle contrast imaging (LSCI) perfusion maps for (**A**) Intact group (*n* = 3); (**B**) Mild severity group (*n* = 7); (**C**) Moderate severity group (*n* = 7); (**D**) Severe severity group (*n* = 2). (**E**) Quantification of the difference in mean perfusion (Δ perfusion units) between hemispheres. (**F**–**I**) Representative 2,3,5-triphenyltetrazolium chloride (TTC)-stained coronal brain sections for (**F**) intact (*n* = 3), (**G**) mild (*n* = 10), (**H**) moderate (*n* = 9), and (**I**) severe (*n* = 2) injury groups. (**J**) Quantification of the hemispheric area difference (in pixels) based on TTC staining sections. Group sizes reflect post hoc stratification based on perfusion severity. Statistical significance was assessed using Kruskal–Wallis or Welch’s ANOVA with appropriate post hoc tests ****—*p* < 0.0001, compared with the intact group, ***—*p* = 0.0005, **—*p* = 0.002.

Seven days after hypoxia–ischemia, cerebral perfusion and macroscopic brain injury were reassessed in animals stratified by early LSCI-based severity classification. Representative LSCI perfusion maps are shown in Figure 3A–D.

Quantitative analysis demonstrated significant differences in Δ mean perfusion among intact, mild, moderate, and severe injury groups (Welch’s ANOVA, W(3, 3.753) = 114.7, *p* < 0.0001; Figure 3E). Post hoc Games–Howell testing revealed significant differences between intact animals and all injury severity groups (*p* < 0.0001), whereas differences between adjacent injury categories were not statistically significant.

Macroscopic assessment of brain injury using TTC staining at day 7 confirmed a stepwise increase in lesion size with increasing injury severity (Figure 3F–I).

Statistically significant differences were observed among the experimental groups: in the severe group—(18,150 ± 1202 px), moderate severity—(5878 ± 353.1 px), mild severity—(3741 ± 466.9 px), in the intact group—(570.3 ± 75.43 px). Welch’s ANOVA revealed a significant overall group effect (W(3, 4.141) = 617.7, *p* < 0.0001; Figure 3J), with post hoc analysis demonstrating significant differences between intact and all injury groups (*p* < 0.0001).

Due to wound healing and scalp regeneration following the early postoperative imaging procedure, repeated LSCI measurements at day 7 were not feasible in a subset of animals from the mild and moderate groups. In addition, the small number of surviving animals in the severe group limited statistical power for within-group correlation analyses at this time point. Therefore, correlation analyses were restricted to pooled group-level comparisons.

## 4. Discussion

The present study demonstrates that early postoperative LSCI enables rapid, noninvasive, and objective stratification of injury severity in a neonatal hypoxia–ischemia model. Although LSCI has been widely validated for monitoring cerebral blood flow dynamics in experimental stroke models [13,14,15,16,17], its use specifically for early post hoc cohort-level stratification in neonatal HI has not been systematically investigated. Our findings address this gap by showing that a single LSCI measurement obtained 3 h after HI can delineate distinct severity categories that reliably predict subsequent macroscopic tissue damage at 24 h and 7 days and survival outcomes. This approach directly responds to the critical need for tools that reduce inter-individual variability, a major factor limiting reproducibility and statistical power in preclinical neonatal HI studies [6,9].

The validity of perfusion-based classification was supported by differences between severity categories in both macroscopic tissue damage and mortality outcomes. These data suggest that stratification based on early perfusion parameters captures biologically meaningful aspects of hypoxic–ischemic injury severity. Incorporating early perfusion assessment may reduce within-group variability and increase sensitivity to detect treatment effects in preclinical neonatal hypoxia–ischemia studies, particularly when therapeutic windows are narrow and sample sizes are limited, conditions under which uncontrolled heterogeneity can obscure therapeutically relevant changes.

From a methodological perspective, these findings highlight a potential avenue to improve experimental design. Identification of animals with comparable injury severity shortly after hypoxia–ischemia allows more appropriate group allocation, reduces outcome variability, and increases statistical power to detect therapeutic effects. When planning neuroprotective intervention studies, extreme phenotypes should be considered separately. Animals without objectively detectable injury (no-visible-damage) should be excluded from subsequent evaluations of neuroprotection, as their inclusion increases the risk of false-positive conclusions regarding treatment efficacy due to the absence of baseline injury and limited variability in perfusion parameters. Likewise, animals with severe injury should be considered at increased risk of false-negative outcomes: extensive damage, high mortality, and limited reversibility may reduce the likelihood of detecting treatment effects even when the intervention is biologically active.

Several limitations and methodological issues of the present study should be acknowledged. First, the perfusion thresholds identified here are specific to the experimental conditions used and should not be regarded as universally applicable across different mouse strains, ages, or hypoxia–ischemia protocols. Second, exclusion of no-visible-damage animals from delayed analyses may limit the generalizability of the findings to milder injury phenotypes. Third, repeated LSCI measurements at later time points were not feasible in all animals due to wound healing and scalp regeneration following the early imaging procedure, which constrained longitudinal assessment of cerebral perfusion dynamics. Fourth, methodological errors occurred during TTC staining, resulting in non-identical staining patterns across brain sections; however, these issues did not affect the qualitative illustration of lesion extent.

We are not the first to propose and illustrate the concept of post hoc stratification of animals after induction of hypoxic–ischemic encephalopathy (HIE) to improve the precision of preclinical studies. However, in the study by MinGi Kim and colleagues, animals without detectable injury are excluded one week after HIE induction, followed by a dichotomous classification into injured versus non-injured groups. While this approach undoubtedly increases the accuracy of outcome assessment and evaluation of post-injury correction, it does not enable a robust assessment of therapeutic interventions initiated on the first day after model induction [20].

Future studies should examine correlations between LSCI-based stratification and other noninvasive biomarkers, such as electrophysiological or metabolic readouts, and evaluate its utility in the context of neuroprotective interventions. Overall, the present results support the use of LSCI as a practical and informative tool for severity-based stratification in neonatal hypoxia–ischemia models, with the potential to improve reproducibility and methodological rigor in preclinical research focused on interventions for neonatal hypoxic–ischemic encephalopathy.

## Data Availability

The original contributions presented in this study are included in the article. Further inquiries can be directed to the corresponding author.

## Abbreviations

The following abbreviations are used in this manuscript:

LSCI	Laser speckle contrast imaging
HI	Neonatal hypoxia–ischemia
NVD	No-visible-damage
TTC	2,3,5-triphenyltetrazolium chloride
